# Supplementary material for: The Needs of Adolescents and Young Adults with Chronic Illness: Results of a Quality Improvement Survey
Source: Children (Basel). 2022 Apr 2;9(4):500. doi: 10.3390/children9040500 (PMC9025253; doi:10.3390/children9040500)
Supplement: Supplementary file 1 [file children-09-00500-s001.zip › Adolescents Survey.pdf]

NIH Needs Survey - Adolescents (13-17 years old)

Age: ______

Sex (please check):
Male Female

Medical Diagnosis: ________________________

Race (please check):
White
Black or African American
Asian
Unknown
American Indian or Alaska Native
Native Hawaiian or Other Pacific Islander
Multi-Race
Other please specify: _____________

Ethnicity (please check):
Hispanic or Latino Not Hispanic or Latino Unknown

Below are areas that adolescents with a health condition may have questions about or need help with. <u>Please check "yes" to any items below that you or others your age would like information about or help with.</u> This information will guide us in planning services to fit the needs of teenagers at the NIH.

HEALTH AND WELLNESS YES

- My health condition
- Medical treatments and side effects
- Nutrition and/or healthy eating
- Physical activity and exercise
- Complementary healthcare (e.g., yoga, meditation)
- My ability to have children in the future

MANAGING PHYSICAL SYMPTOMS YES

- Pain
- Sleep difficulties
- Eating and/or weight (loss or gain)
- Appearance (how I look)
- Physical limitations (e.g., difficulties walking, poor vision, problems hearing)

SOCIAL-EMOTIONAL WELLBEING YES

- Symptoms and treatment of anxiety or depression
- Coping better with my medical condition and treatment
- Relaxation/stress management tools
- Getting support from my family or friends
- Talking to others about my medical condition
- Keeping and making friends
- Dating, relationships, sexual identity
- Uncertainty about the future

LEARNING, SCHOOL, AND WORK YES

- Learning difficulties
- Attention problems
- Returning to school after treatment
- Transitioning to college
- Jobs/careers that fits my skills/abilities
- Impact of my medical condition at school or work

ADJUSTMENT DURING AND AFTER TREATMENT YES

- Adjusting to my diagnosis and treatment at NIH
- Communicating with my healthcare providers throughout treatment (e.g., learning to speak up about my needs or ask questions about treatment)
- Adjusting to life after treatment (e.g., fitting back in with friends, moving on with my life)
- Having difficult conversations (e.g., talking about my diagnosis, prognosis, or concerns related to death and dying)

HEALTHCARE AND PRACTICAL NEEDS YES

- Transitioning from pediatric care to adult care
- Medical insurance (Medicaid/private)
- Legal and practical services (e.g., living will, power of attorney)

NIH Needs Survey - Adolescents (13-17 years old)

Below are services, events, or programs that may be helpful for adolescents at the NIH. Please check "yes" to any items below that you or others your age would be interested in to guide us in planning services for teens at the NIH:

COUNSELING SERVICES THROUGH NIH

YES

- Supportive counseling for myself while I am at NIH
- Supportive counseling for my family while at NIH
- One-on-one video chat sessions at home to check in with a NIH therapist in between visits
- Attending in-person support groups for teens with my health condition at NIH
- Participating in an online support group or chat room for teens with my health condition

NIH GROUPS AND EVENTS

YES

- A NIH electronic newsletter that provides information related to my health condition
- A NIH website that provides information related to my health condition
- Connecting with others who have a similar health condition through social media (e.g., Facebook, Twitter)
- A NIH event where experts would address issues that teens face related to my health condition
- Would you be willing to make an extra visit to NIH to attend an event for patients or families?

RECREATIONAL SERVICES THROUGH NIH

YES

- A hang-out space at NIH for teenagers
- Recreational weekend programs with other teens with the same/similar health condition
- Scheduled recreational activities during the day for teens (e.g., games, bowling)

Please write in your comments to give us additional information and your ideas:

WHAT ARE THE TOP 3 THINGS THAT WOULD IMPROVE YOUR EXPERIENCE AT THE NIH?

1. ______________________________

2. ______________________________

3. ______________________________

PLEASE SHARE OTHER IDEAS OR COMMENTS YOU HAVE TO HELP US MAKE NIH BETTER FOR TEENS!

____

____

____

____

____
