# Supplementary material for: The Needs of Adolescents and Young Adults with Chronic Illness: Results of a Quality Improvement Survey
Source: Children (Basel). 2022 Apr 2;9(4):500. doi: 10.3390/children9040500 (PMC9025253; doi:10.3390/children9040500)
Supplement: Supplementary file 1 [file children-09-00500-s001.zip › Parents Survey.pdf]

NIH Needs Survey - Parents/Caregivers of Adolescents and Young Adults (13-35 years old)

Child's Age: ______ **Caregiver Age:** ______

Child's Sex (please check): Male Female

Caregiver's Sex (please check): Male Female

Child's Primary Medical Diagnosis:

____

Caregiver Race (please check):

White | American Indian or Alaska Native
Black or African American | Native Hawaiian or Other Pacific Islander
Asian | Multi-Race
Unknown | Other please specify: ____________

Caregiver Ethnicity (please check):

Hispanic or Latino | Not Hispanic or Latino | Unknown

The first section of this form asks about things that your child may need, while the second section asks about your needs as a parent. This information will guide us in planning services to fit the needs of patients and caregivers at the NIH.

Section 1. In this section, please check "yes" for any items that you believe your child (or others his/her age) would like information about, help with, or participate in while at the NIH.

CHILD'S HEALTH

YES

- His or her health condition
- Medical treatments and side effects
- Nutrition and/or healthy eating
- Physical activity and exercise
- Complementary healthcare (e.g., yoga, meditation)
- His/her ability to have children in the future

CHILD'S PHYSICAL SYMPTOMS

YES

- Pain
- Sleep difficulties
- Eating and/or weight (loss or gain)
- Appearance
- Physical limitations (e.g., difficulties walking, poor vision, problems hearing)

CHILD'S SOCIAL-EMOTIONAL WELLBEING

YES

- Symptoms and treatment of anxiety or depression
- Coping with his/her medical condition and treatment
- Relaxation/stress management tools
- Getting support from his/her family or friends
- Talking to others about his/her medical condition
- Keeping and making friends
- Dating, relationships, sexual identity
- Uncertainty about the future

CHILD'S LEARNING, SCHOOL, AND WORK

YES

- Learning difficulties
- Attention problems
- Returning to school after treatment
- Transitioning to college
- Jobs/careers that fit his/her skills/abilities
- Impact of his/her medical condition at school or work

CHILD'S ADJUSTMENT DURING OR AFTER TREATMENT

YES

- Adjusting to his/her diagnosis and treatment at NIH
- Communicating with his/her healthcare providers throughout treatment (e.g., learning to speak up about needs or ask questions about treatment)
- Adjusting to life after treatment (e.g., fitting back in with friends, moving on with his/her life)
- Having difficult conversations (e.g., talking about his/her diagnosis, prognosis, or concerns related to death and dying)

CHILD'S HEALTHCARE AND PRACTICAL NEEDS

YES

- Transitioning from pediatric care to adult care
- Medical insurance (Medicaid/private)
- Legal and practical services (e.g., living will, power of attorney)

COUNSELING SERVICES AT NIH FOR YOUTH

YES

- Supportive counseling while my child is at NIH
- One-on-one video chat sessions at home for my child to check in with a NIH therapist between visits
- Attending in-person support groups for teens/ young adults with similar health conditions at NIH
- Participating in an online support group or chat room for teens/young adults with a similar health condition

RECREATIONAL SERVICES FOR YOUTH THROUGH NIH

YES

- A hang-out space at NIH for youth
- Recreational weekend programs with other youth with the same/similar health condition
- Scheduled recreational activities during the day for youth (e.g., games, bowling)

NIH GROUPS AND EVENTS FOR YOUTH

YES

- A NIH electronic newsletter that provides information related to your child's health condition
- A NIH website that provides information related to your child's health condition
- Connecting your child with others who have a similar health condition through social media (e.g., Facebook, Twitter)
- A NIH event for youth where experts would address issues that youth face related to their health condition

NIH Needs Survey - Parents/Caregivers of Adolescents and Young Adults (13-35 years old)

<u>Section 2.</u> In this section, please check "yes" for any items that <u>you or other caregivers</u> would like information about, help with, or to participate in at the NIH to guide us in planning services for caregivers at the NIH:

GAINING KNOWLEDGE ABOUT MY CHILD'S HEALTH — YES

- Condition-specific information (e.g., leukemia, NF1)
- Nutrition and/or healthy eating
- Physical activity and exercise
- Sleep habits
- Complementary medicine for my child (e.g., yoga, meditation, acupuncture)
- Transition from pediatric care to adult care
- Fertility issues for my child

UNDERSTANDING AND HELPING MY CHILD COPE WITH: — YES

- Physical pain
- Unpleasant side effects of treatment
- Anxiety and/or depression
- Attention difficulties
- Learning/academic functioning
- General stress in the context of his/her medical condition
- Social relationships with his/her peers

SOCIAL-EMOTIONAL WELLBEING OF <u>CAREGIVERS</u> — YES

- Symptoms and treatment of anxiety or depression
- Relaxation/stress management tools
- Spiritual wellbeing and/or spiritual/religious coping
- Self-care
- Getting support from family or friends
- Maintaining friendships or relationships
- Talking to others about my child's medical condition
- Talking to my child about his/her prognosis
- Talking to my child if treatment is not working

FINANCIAL AND FAMILY NEEDS — YES

- Medical insurance (Medicaid/private)
- Legal and practical services (e.g., living will, power of attorney, advance care planning)
- Identifying financial resources while my child is undergoing treatment
- Caring for my other children while managing the unique health needs of my child at NIH

COUNSELING SERVICES <u>FOR CAREGIVERS</u> AT NIH — YES

- Supportive counseling to cope with my child's medical condition and treatment while at NIH
- Marital/couples counseling
- One-on-one video chat sessions at home to check in with a NIH therapist in between my child's visits
- Attending in-person support groups with other caregivers of youth facing a similar health condition
- Participating in an online support group or chat room with other caregivers of youth facing a similar health condition

NIH GROUPS AND EVENTS — YES

- A NIH electronic newsletter that provides information related to my child's health condition
- A NIH website that provides information related to my child's health condition
- Connecting with other caregivers of children with a similar health condition through social media (e.g., Facebook, Twitter)
- A NIH event where experts would address issues that families face in the context of chronic/acute health conditions
- Would you/your child be willing to make an extra visit to NIH to attend an event for patients or families?

Please write in your comments to give us additional information and your ideas:

WHAT ARE THE TOP 3 THINGS THAT WOULD IMPROVE YOUR EXPERIENCE AT THE NIH?

1. ______________________________

2. ______________________________

3. ______________________________

PLEASE SHARE OTHER IDEAS OR COMMENTS YOU HAVE TO HELP US MAKE NIH BETTER FOR PATIENTS AND CAREGIVERS!

____

____

____
