# Supplementary material for: The Needs of Adolescents and Young Adults with Chronic Illness: Results of a Quality Improvement Survey
Source: Children (Basel). 2022 Apr 2;9(4):500. doi: 10.3390/children9040500 (PMC9025253; doi:10.3390/children9040500)
Supplement: Supplementary file 1 [file children-09-00500-s001.zip › Young Adult Survey.pdf]

NIH Needs Survey - Young Adults (18-35 years old)

Age: ______

Sex (please check):
Male Female

Primary Medical Diagnosis:
____

Race (please check):
White
Black or African American
Asian
Unknown
American Indian or Alaska Native
Native Hawaiian or Other Pacific Islander
Multi-Race
Other please specify: _____________

Ethnicity (please check):
Hispanic or Latino Not Hispanic or Latino Unknown

Below are areas that young adults with a health condition may have questions about or need help with. Please check "yes" to any items below that you or others your age would like information about or help with. This information will guide us in planning services to fit the needs of young adults at the NIH.

HEALTH AND WELLNESS

YES

- My health condition
- Medical treatments and side effects
- Nutrition and/or healthy eating
- Physical activity and exercise
- Complementary health practices (e.g., yoga, meditation)
- Fertility (my ability to have children)

MANAGING PHYSICAL SYMPTOMS

YES

- Pain
- Sleep difficulties
- Eating and/or weight (loss or gain)
- Appearance (how I look)
- Physical limitations (e.g., difficulties walking, poor vision, problems hearing)

SOCIAL-EMOTIONAL WELLBEING

YES

- Symptoms and treatment of anxiety or depression
- Coping with my medical condition and treatment
- Relaxation/stress management tools
- Spiritual wellbeing and spiritual/religious coping
- Getting the support I need from my family or friends
- Talking to others about my medical condition
- Keeping and making friends
- Dating, sexuality, and intimacy
- Coping with uncertainty about the future

SCHOOL OR WORK

YES

- Learning difficulties
- Attention problems
- Jobs/careers that fit my skills and abilities
- Academic support in college
- Transitioning back to school or work after treatment
- Impact of my medical condition at school or work

ADJUSTMENT DURING AND AFTER TREATMENT

YES

- Adjusting to my diagnosis and treatment at NIH
- Communicating with my healthcare providers throughout treatment (e.g., learning to speak up about my needs or ask questions about treatment)
- Adjusting to life after treatment (e.g., fitting back in with friends, moving on with my life)
- Having difficult conversations (e.g., talking about my diagnosis, prognosis, or concerns related to death and dying)

HEALTHCARE AND FINANCIAL NEEDS

YES

- Medical insurance (Medicaid/private)
- Legal and practical services (e.g., living will, advance care planning)
- Financial assistance while undergoing treatment
- Finding a new doctor as an adult

NIH Needs Survey - Young Adults (18-35 years old)

Below are services, events, or programs that may be helpful for young adults at the NIH. Please check "yes" to any items below that you or others your age would be interested in to guide us in planning services for young adults at the NIH:

COUNSELING SERVICES	YES
• Supportive counseling for myself while I am at NIH	
• Supportive counseling for my family (e.g., my parents, my spouse) while we are at NIH	
• One-on-one video chat sessions at home to check in with a NIH therapist in between visits	
• Attending in-person support groups for young adults with my health condition at NIH	
• Participating in an online support group or chat room for young adults with my health condition	

NIH GROUPS/EVENTS	YES
• A NIH electronic newsletter that provides information related to my health condition	
• A NIH website that provides information related to my health condition	
• Connecting with others who have a similar health condition through a social media group (e.g., Facebook, Twitter)	
• A NIH event where experts would address issues that young adults face related to their health condition	
• Would you be willing to make an extra visit to NIH to attend an event for patients or families?	

RECREATIONAL SERVICES THROUGH NIH	YES
• A hang-out space at NIH for young adults	
• Recreational weekend programs with other young adults with the same/similar health condition	
• Scheduled recreational activities during the day for young adults (e.g., games, bowling)	

Please write in your comments to give us additional information and your ideas:

WHAT ARE THE TOP 3 THINGS THAT WOULD IMPROVE YOUR EXPERIENCE AT THE NIH?

1. ______________________________

2. ______________________________

3. ______________________________

PLEASE SHARE OTHER IDEAS OR COMMENTS YOU HAVE TO HELP US MAKE NIH BETTER FOR YOUNG ADULTS!

____

____

____

____

____
